# Impact of incorrect tissue classification in Dixon-based MR-AC: fat-water tissue inversion

**DOI:** 10.1186/s40658-014-0101-0

**Published:** 2014-12-14

**Authors:** Claes Nøhr Ladefoged, Adam Espe Hansen, Sune Høgild Keller, Søren Holm, Ian Law, Thomas Beyer, Liselotte Højgaard, Andreas Kjær, Flemming Littrup Andersen

**Affiliations:** Department of Clinical Physiology, Nuclear Medicine and PET, Rigshospitalet, University of Copenhagen, Blegdamsvej 9, 2100 Copenhagen, Denmark; Centre for Medical Physics and Biomedical Engineering, Medical University of Vienna, Waehringer Guertel 18-20/4L, Vienna, A-1090 Austria

**Keywords:** PET/MR, Fat-water inversion, Image artifacts, DWFS

## Abstract

**Background:**

The current MR-based attenuation correction (AC) used in combined PET/MR systems computes a Dixon attenuation map (MR-AC_Dixon_) based on fat and water images derived from in- and opposed-phase MRI. We observed an occasional fat/water inversion in MR-AC_Dixon_. The aim of our study was to estimate the prevalence of this phenomenon in a large patient cohort and assess the possible bias on PET data.

**Methods:**

PET/MRI was performed on a Siemens Biograph mMR (Siemens AG, Erlangen, Germany). We visually inspected attenuation maps of 283 brain or head/neck (H/N) patients, classified them as non-inverted or inverted, and calculated the fat/water tissue fraction. We selected ten FDG-PET brain patients with non-inverted attenuation maps for further analysis. Tissue inversion was simulated, and PET images were reconstructed using both original and inverted attenuation maps. The FDG-PET images of the ten brain patients were analyzed using 11 concentric annulus regions of 5 mm width placed over a central transaxial image plane traversing PET_Dixon_.

**Results:**

Out of the 283 patients, a fat/water inversion in 23 patients (8.1%) was observed. The average fraction of fat in the correct MR-AC_Dixon_ was 13% for brain and 17% for H/N patients. In the inverted cases, we found an average fat fraction of 56% for the brain patients and 41% for the H/N patients. The effect of the simulated tissue inversion in the brain studies was clearly seen on AC-PET images. The percent-difference image revealed a radial error where the largest difference was at the ventricles (30% ± 3%) and smallest at the cortical region (10% ± 2%).

**Conclusions:**

Tissue inversion in Dixon MRI is well known and can occur when there is an error in the off-resonance correction method. Tissue inversion needs to be considered if, based on Dixon-AC, the construction of normal PET databases is performed or any quantitative physiological parameters are fitted. Visual inspection is needed if Dixon-AC is to be used in clinical routine.

## Background

Integrated PET/MR is an area of rapidly growing interest in the medical community. One challenge still to overcome is the absence of CT-like transmission sources in combined clinical PET/MR systems to perform attenuation correction (AC). Unlike the Hounsfield units of CT data that are directly related to the linear attenuation coefficients (LACs) of the 511 keV photons in PET, MRI is a function of proton density and tissue relaxation times that have no direct relation to the LACs.

**Figure 1:**
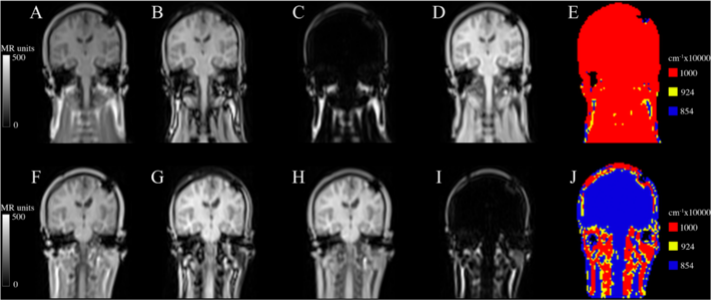
**Dixon images in a patient with both inverted and non-inverted fat/water tissues.** In the first scan **(A-E)** and inverted in second scan **(F-J)**. **(A)** and **(F)** in-phase, **(B)** and **(G)** opposed-phase, **(C)** and **(H)** fat, **(D)** and **(I)** water, and **(E)** and **(J)** MR-AC_Dixon_ shown in rainbow colors for visualization purposes.

Different approaches have been suggested to calculate segmented AC maps. The Philips Ingenuity TF PET/MR (Philips International B.V., Amsterdam, The Netherlands) uses a T1-weighted image acquisition for three-class tissue separation (air, lung, tissue) and body delineation [1], whereas the Siemens Biograph mMR (Siemens AG, Erlangen, Germany) uses a two-point Dixon MR sequence to separate the tissues in four classes (air, lung, fat and soft tissue) [2-4]. Thresholds applied to the water and fat images are used to identify voxels that correspond to fat and soft tissue and to separate them from the background; voxels representing a mixture of fat and soft tissue were also used by Martinez-Möller et al. [4] when the values in both images were above these thresholds, this is illustrated in yellow in Figure 1E.

The MR signal is dominated by components arising from protons in water and lipid (fat), which have different resonance frequencies. A two-point Dixon sequence consists of a *0 image* with fat and water in-phase (Figure 1A) and a π *image* with fat and water out-phase (Figure 1B) to obtain chemical shift sensitive images. Water or fat only images can be obtained from the phase images [2,3] (Figure 1C,D). The largest signal in the water or fat images may be used to decide the classified tissue in each voxel.

A number of errors in the attenuation maps have been reported since the introduction of combined PET/MR [5], including tissue inversion where the fat and water classes are incorrectly identified under phase unwrapping [2,6,7]. This results in the water image looking like a normal fat image and the fat like a normal water image. As the current attenuation correction used in the combined mMR derives the attenuation map (MR-AC_Dixon_) from fat and water images based on the Dixon MR [4], a global incorrect assignment of LACs will occur in these cases. Incorrect assignment of LACs can lead to large errors in estimated PET activity if LACs are misclassified or simply under- or overestimated [8]. Here, we evaluate the prevalence of fat-water tissue inversion on the Siemens mMR in a large patient population of head and neck patients and assess the effect on the PET images after attenuation correction.

## Methods

### Imaging protocol

PET/MRI was performed on a fully integrated Siemens mMR Biograph [9]. All patients were positioned headfirst, with their arms down, and data were acquired over a single bed position of 25.8 cm. Standard MR-based attenuation maps (MR-AC_Dixon_) were derived using the Dixon VIBE sequence [4], as implemented by the vendor. The Dixon-water image and the MR-AC attenuation maps were reconstructed on 192 × 126 × 128 matrices (2.6 × 2.6 × 3.1 mm voxels).

### Patients

Datasets were gathered retrospectively from 283 brain or head/neck (H/N) patients (120 F, 163 M) acquired since the system installation at our center, and a manual inspection was performed. Each attenuation map was classified as non-inverted or inverted, and the tissue-fraction distribution

1 $$ {\mathrm{fraction}}_X = \frac{\#\ {\mathrm{Voxels}}_X}{\#{\mathrm{Voxels}}_{\mathrm{FAT}{\displaystyle \cup \mathrm{WATER}}}}\times 100\% $$

was calculated, where *X* is fat or water. We report these results, as well as the averaged weight and age, for all brain and H/N patients.

Of the 283 patients, we selected a subgroup of 17 patients scanned with [^18^F]-F-fluoro-ethyl-tyrosine (FET) that had at least two scans due to therapy follow-up and inspected their attenuation maps for tissue inversion.

### Simulated tissue inversion and the effect on PET images

A subset of ten patients from the original 283 referred for [^18^F]-fluorodeoxyglucose (FDG) PET brain scanning for suspected dementia were selected for analysis (age: (63 ± 15) years; weight: (71 ± 14) kg; injected dose: (197 ± 3) MBq; post injection time: 50.3 ± 9.9 min). The patients all had non-inverted attenuation maps (MR-AC_Dixon_) and fat/water inversion (MR-AC_Inverted_) was simulated by setting all voxels with *μ* = 0.1 cm^−1^ (water) to 0.0854 cm^−1^ (fat) and vice versa. The PET images were reconstructed with and without MR-AC using OP-OSEM (four iterations, 21 subsets, 3 mm Gaussian post filtering) on 344 × 344 matrices (2.1 × 2.1 × 2.0 mm voxels) into the resulting PET_Dixon_ and PET_Inverted_.

The FDG-PET brain images of the ten patients were further analyzed using one central transaxial image plane traversing PET_Dixon_. Eleven concentric annular regions of interest of 5 mm width were drawn, starting from the edge of the cortex, and the mean activity (kBq/mL) was calculated within all annuli, as in [10]. The relative percent differences were calculated as follows:

2 $$ \varDelta \% = \frac{{\mathrm{PET}}_{\mathrm{Dixon}}-{\mathrm{PET}}_{\mathrm{Inverted}}}{{\mathrm{PET}}_{\mathrm{Dixon}}}\times 100 $$

This was done to capture radial differences in errors, based on the expectation that a global underestimation of tissue will result in the largest error in the center of the brain [10]. We report the tissue-fraction distributions and Δ% difference for each of the regions and patients.

### Case study: effect outside the head and neck region

**Figure 2:**
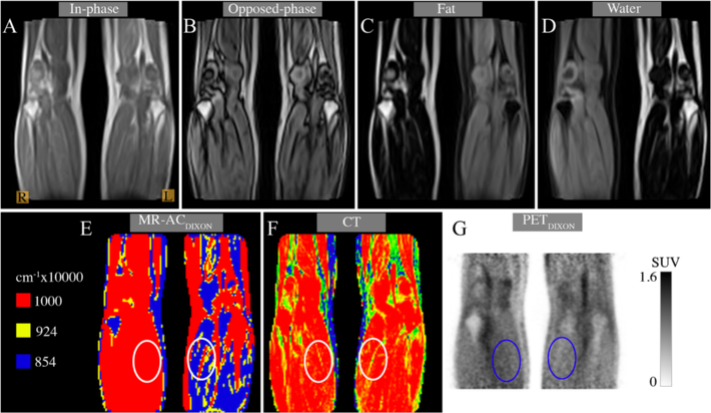
**Patient with one fat/water inverted leg (patient left) and one non-inverted (patient right).** **(A)** In-phase and **(B)** opposed-phase images. Notice only the left leg has inverted values **(C-E)**. ROI delineation of anatomically matched reference regions drawn on **(E)** and transposed onto **(F)** and **(G)**. Mean values for right/left leg are 0.1/0.09, 0.099/0.099 cm^−1^, 0.595/0.412 SUV, for **(E-G)**, respectively.

As a case study, we included a patient with a regional scan covering the lower hamstrings to the calf muscles (age: 60 years; weight: 85 kg; injected dose: 340 MBq; post injection time: 151 min). The patient presented with tissue inversion in one leg only (Figure 2). We aligned the CT image of the patient using minctracc (McConnell Imaging Center, Montreal) to obtain an attenuation map without tissue inversion. PET images were reconstructed with the original MR-AC_Dixon_ using the same parameters as the previous sub-section, only changing the number of iterations to 3 and Gaussian post filtering to 4 mm. We placed region of interests (ROIs) in anatomically matched reference regions in each leg and measured the mean tracer uptake in the PET image reconstructed using Dixon, as well as the one reconstructed using the aligned CT.

## Results

**Figure 3:**
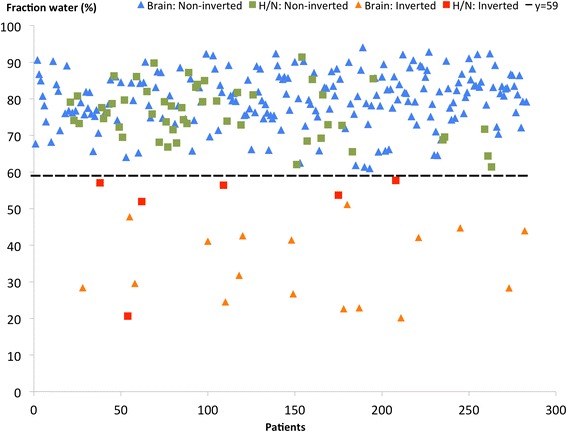
**Distribution of tissue fractions.** We show, for each patient, the fraction of water pixels to the total number of tissue pixels in the attenuation map. Symbol type represents head/neck and brain patients and inverted or non-inverted cases, as shown in the legend. The dashed black line illustrates the split of two groups (non-inverted and inverted) in two non-overlapping parts.

It is straightforward to visually identify an inverted MR-AC_Dixon_ (Figure 1). Out of the 283 included patients, there was an inversion of fat and water attenuation values in 23 patients (8.1%). The average fraction of tissue classified as fat (fraction_Fat_, Equation (1)) in the non-inverted MR-AC_Dixon_ was 13% (±5%; range: 4% to 30%) for brain and 17% (±6; range: 6% to 31%) for H/N patients. In the inverted cases, we found an average fat fraction of 56% (±9%; range: 40% to 69%) for the brain and 41% (±11%; range: 33% to 67%) for the H/N patients. There was no overlap between the inverted and non-inverted groups in the plot (Figure 3), as indicated by the dashed line. The average weight and age for the non-inverted patients were 77 kg and 60 years and 73 kg and 53 years for the inverted. There were no significant differences in the mean weight between the groups (two sample *t* test; *p* > 0.2). The classification results were randomly distributed over time, meaning that it did not coincide with, e.g., system software release.

**Figure 4:**
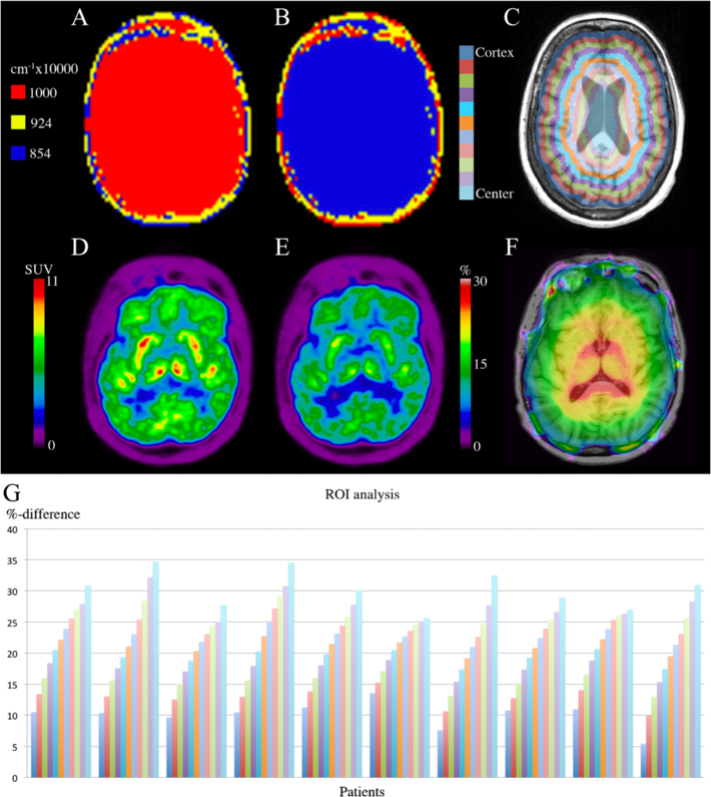
**Effect on PET of simulated fat/water inversion. (A)** MR-AC_Dixon_ and **(B)** MR- AC_Inverted_, **(C)** Annuli drawn on PET_Dixon_ fused onto T1w MPRAGE, **(D)** PET_Dixon_ and **(E)** PET_Inverted_, **(F)** Δ% (PET_Dixon_, PET_Inverted_) fused onto T1w MPRAGE. **(G)** ROI analysis of the 11 annuli drawn on the ten FDG brain patients. Color code corresponds to coloring on **(C)**. Notice the visual differences between the PETs in **(D)** and **(E)** and the radial gradient in **(F)** which corresponds to the results in **(G)**. The patient in **(A-F)** is the third from the left in **(G)**.

For the subgroup of 17 patients with repeat scans (Table 1), two patients had fat/water inversion in both scans and two had inversion in one scan and non-inversion in the other (Figure 1). The result of the simulated fat/water inversion when compared to the original is shown for a single patient in Figure 4 A,B,C,D,E,F. The effect of the inversion can be seen clearly when inspecting the PET images (Figure 4 D,E). The percent-difference image (Figure 4F) displays a radial gradient where the error is largest at the ventricles (35%) and smallest at the cortex (10%). This is the case for all patients (Figure 4G).

**Table 1:** **FET-PET repeat scan results**

	**Number of patients**
Both scans non-inverted	13
Both scans inverted	2
One scan non-inverted, other inverted	2

A sample patient from the last row is shown in Figure 1.

**Figure 5:**
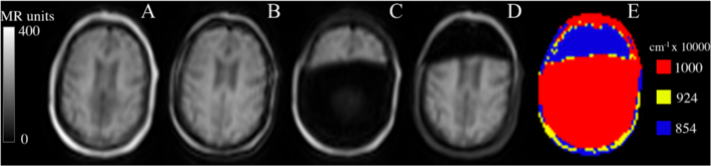
**Dixon images in a patient with inverted fat/water tissues in a subset of the volume. (A)** In-phase, **(B)** opposed-phase, **(C)** fat, **(D)** water, and **(E)** MR-AC_Dixon_.

Fat/water inversion is not only a global event. Inversion in only a part of the image volume was observed on two occasions of which one is shown in Figure 5. Inside the ROI, placed in the coronal slice on the case study patient with inversion in only one leg (Figure 2 E,F,G), the mean attenuation value in the Dixon image was 10% lower in the inverted leg ROI (*μ* = 0.09 versus 0.1 cm^−1^) while the CT had the same attenuation value in both ROIs (*μ* = 0.099 cm^−1^). This underestimation resulted in 30.8% lower SUV_MEAN_ uptake in the ROI placed in the inverted leg, compared to the non-inverted leg.

## Discussion

The primary findings of the current study are that tissue inversion of water and fat has a not negligible prevalence in Dixon MRI of the brain and head/neck (23 of 283 patients). When Dixon MRI is subsequently used for attenuation correction, tissue inversion has a large quantitative effect on the resulting PET images (up to 35% error and non-uniformly distributed). Quantitative errors of this large magnitude are important for both clinical and research use of PET/MR.

For both brain and H/N patients, there was no overlap in the fraction of water voxels between inverted and non-inverted AC maps (Figure 3). This suggests that the identification of inverted MR-AC_Dixon_ can easily be automated based on the fat fraction in the image. Visual inspection is needed in the case of partial inversions, which were observed only infrequently (2 of 283 patients). We did not analyze the data with respect to anatomical anomalies occurring post surgery or from metallic implants, except for dental implants where no correlation with tissue inversion was found.

To analyze the extent of the error introduced by the fat/water inversion, we chose to use patients without inversion in order to have a gold standard PET image as a fair comparator. The images following the simulated inversion had tissue-fraction distributions similar to the brain patients with actual fat/water inversion, although with a slightly higher fraction of fat. We trust the results to be representative despite this overestimation introduced by the simulation.

The error for a given line-of-response (LOR) depends on the attenuation coefficient error and length of the path through the inverted tissue. The LORs passing the center must on average intersect more misclassified tissue than tangential LORs near the edges. This explains the radial bias observed (Figure 4G). The bias was consistent in all ten patients.

Problems using Dixon MR-based attenuation correction in PET/MR have previously been reported [5], including a case of false tissue assignment to lower thorax where lung tissue is assigned the attenuation coefficient of air. The phenomenon of inversion of water and fat is also well known [4] and occurs due to off-resonance effects caused by inhomogeneity of the B0 magnetic field. Off-resonance effects render the identification of water and fat from in- and opposed-phase images ambiguous, thus, leading to potential inversion artifacts. This ambiguity can be resolved using a number of methods; however, without knowing the exact implementation details, the reason for the fat/water inversion can only be speculated on. The most common method is the region-growing approach where the off-resonance region is determined one voxel at a time starting from one or more seed points. Choosing incorrect seed points might lead to incorrect tissue identification. This might be the explanation in the case imaging the legs (Figure 2 A,B,C,D,E). Multiple seed points are needed since the legs are not connected and choosing incorrect seed point in one leg could explain the inversion. This is further supported by the fact that the in-phase and opposed-phase images (Figure 2A,B) have identical left and right legs, indicating that the error occurs when identifying water and fat images.

The manufacturer's user manual advises that the attenuation maps should be visually inspected prior to continuation of the scan. In the case of an error, such as with tissue inversions, the manual advises a repetition of the scan. However, in our experience (supported by the few repeat examinations reported here), the error can be reproducible, so a rescan does not necessarily provide non-inverted images. For two patients, the inversion was only present in one of the repeat scans. With an underestimation of PET uptake of 10% to 35% in the case of inversion, such cases of inconsistent inversions are of course a significant problem for follow-up examinations and response evaluation which Wahl et al. [11] discussed in the case of solid tumors. Hence, the problem can only be corrected for by an off-line user-implemented inversion of fat and water, as was performed here. It is expected that tissue inversion is more frequent in the case of small body parts. A systematic comparison of the frequency of the effect for different body parts is out of the scope of the present work.

The findings in this paper add further concerns to those previously voiced [5,10,12-14]. Tissue inversion needs to be considered if the construction of normal PET databases is performed based on Dixon-AC or if quantitative measures for simple semiquantitative analysis such as the standardized uptake value (SUV) are being used to set cut-off thresholds or follow treatment effects. We do not recommend the use of Dixon-AC for pharmacokinetic modeling of physiological parameters in the brain [10]. In longitudinal follow-up in, e.g., dementia using FDG, regional activity may increase or decrease and using FET for tumor imaging the metabolically active area may change configuration. The study was limited to screening a large database of head and neck patients. However, the observed tissue swap effect can occur also in areas outside the head and neck as illustrated by the anecdotal example of the tissue inversion in the upper thighs. Therefore, utmost care is needed if Dixon-AC is to be used in clinical routine.

## Conclusions

MR-based attenuation correction is still a challenge. In this paper, we have shown that incorrect identification of fat and water classes has a strong impact on the PET images following AC. We have shown that the inversion occurs frequently and is not predictable. An automatic method to detect fat/water inversions seems possible when the full volume is inverted, but in the case of inversion in only a subset of the slices, a manual inspection of the attenuation maps is required. As the non-inverted attenuation map cannot be calculated directly from the inverted, it is important to inspect the attenuation map if the PET images should be used quantitatively. In the brain, this might also be the case even if the PET images are only read visually as indicated in our simulated inversion study.

## References

[1] Schulz V, Torres-Espallardo I, Renisch S, Hu Z, Ojha N, Börnert P, Perkuhn M, Niendorf T, Schäfer WM, Bockmann H, Krohn T, Buhl A, Günther RW, Mottaghy FM, Krombach GA (2011). Automatic, three-segment, MR-based attenuation correction for whole-body PET/MR data. Eur J Med Mol Imaging.

[2] Coombs BD, Szumowski J, Coshow W (1997). Two-point Dixon technique for water-fat signal decomposition with B0 inhomogeneity correction. Magn Reson Med.

[3] Dixon WT (1984). Simple proton spectroscopic imaging. Radiology.

[4] Martinez-Möller A, Souvatzoglou M, Delso G, Bundschuh RA, Chefd'hotel C, Ziegler SI, Navab N, Schwaiger M, Nekolla SG (2009). Tissue classification as a potential approach for attenuation correction in whole-body PET/MRI: evaluation with PET/CT data. J Nucl Med.

[5] Keller SH, Holm S, Hansen AE, Sattler B, Andersen F, Klausen TL, Højgaard L, Kjær A, Beyer T (2013). Image artifacts from MR-based attenuation correction in clinical, whole-body PET/MRI. MAGMA.

[6] Ma J (2008). Dixon techniques for water and fat imaging. J Magn Reson Imaging.

[7] Keller SH, Hansen AE, Holm S, Beyer T, Carrio I, Ros P (2014). Image distortions in clinical PET/MR imaging. PET/MRI.

[8] Huang SC, Hoffman EJ, Phelps ME, Kuhl DE (1979). Quantitation in positron emission computed tomography: 2. Effects of inaccurate attenuation correction. JCAT.

[9] Delso G, Fürst S, Jakoby B, Ladebeck R, Ganter C, Nekolla SG, Schwaiger M, Ziegler SI (2011). Performance measurements of the Siemens mMR integrated whole-body PET/MR scanner. J Nucl Med.

[10] Andersen FL, Ladefoged CN, Beyer T, Keller SH, Hansen AE, Højgaard L, Kjær A, Law I, Holm S (2014). Combined PET/MR imaging in neurology: MR-based attenuation correction implies a strong spatial bias when ignoring bone. Neuroimage.

[11] Wahl RL, Jacene H, Kasamon Y, Lodge MA (2009). From RECIST to PERCIST: evolving considerations for PET response criteria in solid tumors. J Nucl Med.

[12] Hitz S, Habekost C, Fürst S, Delso G, Förster S, Ziegler S, Nekolla SG, Souvatzoglou M, Beer AJ, Grimmer T, Eiber M, Schwaiger M, Dzezga A (2014). Systematic comparison of the performance of integrated whole-body PET/MR imaging to conventional PET/CT for 18F-FDG brain imaging in patients examined for suspected dementia. J Nucl Med.

[13] Dickson JC, O'Meara C, Barnes A (2014). A comparison of CT- and MR-based attenuation correction in neurological PET. Eur J Nucl Med Mol Imaging.

[14] Ladefoged CN, Andersen FL, Keller SH, Löfgren J, Hansen AE, Holm S, Højgaard L, Beyer T (2013). PET/MR imaging of the pelvis in the presence of endoprostheses: reducing image artifacts and increasing accuracy through inpainting. Eur J Nucl Med Mol Imaging.

